# Bio-Inspired Metaheuristic Optimization of a DWT–BiLSTM Architecture for Wind Speed Forecasting: A Statistical Benchmark with Component Ablation

**DOI:** 10.3390/biomimetics11080568

**Published:** 2026-08-08

**Authors:** Emre Bendeş

**Affiliations:** Department of Computer Engineering, Engineering—Architecture Faculty, Nevşehir Hacı Bektaş Veli University, 50300 Nevşehir, Türkiye; emrebendes@nevsehir.edu.tr

**Keywords:** bio-inspired computation, swarm intelligence, metaheuristic optimization, wind speed forecasting, discrete wavelet transform, bidirectional LSTM, hyperparameter optimization, Grey Wolf Optimizer, exploration–exploitation

## Abstract

Population-based bio-inspired metaheuristics are the dominant tools for tuning hybrid decomposition–deep-learning forecasters, yet their relative behavior on a common problem is rarely assessed with a leakage-free, physically meaningful protocol. We benchmark eight metaheuristics on the joint nine-dimensional hyperparameter optimization of a discrete-wavelet-transform bidirectional-LSTM (DWT–BiLSTM) architecture for short-term wind speed forecasting, using 409,152 hourly observations from eight meteorological stations. The set comprises six nature-inspired methods (Artificial Bee Colony, ABC; genetic algorithm, GA; Particle Swarm Optimization, PSO; Grey Wolf Optimizer, GWO; Hippopotamus Optimization, HO; and the Raindrop Optimizer) together with two recent metaphor-free or social variants (the Farthest-better Nearest-worse Optimizer, FNO; and the Tuckman Optimization Algorithm, TOA). A multi-stage protocol covers 30 independent runs per algorithm, a joint-versus-sequential comparison, a genuine rolling-origin out-of-sample evaluation, and component ablation. Friedman testing reveals significant differences (χ^2^ = 49.76; *p* < 10^−8^), with the Grey Wolf Optimizer attaining the best mean rank (2.27) and Pareto-dominant run-time; ablation shows the DWT front-end is essential (Cohen’s d = 13.09) and bidirectionality negligible at the one-hour horizon (*p* = 0.674). Critically, evaluating forecasts in reconstructed physical units reveals that the per-component advantage does not persist: at the one-hour horizon the reconstructed forecast does not exceed a naive persistence baseline (skill ≈ −0.5 in m/s versus +0.44 in normalized component space), a discrepancy independent of decomposition leakage that we report transparently. This work thus contributes a rigorous, leakage-controlled bio-inspired benchmark and a cautionary evaluation methodology.

## 1. Introduction

Wind energy has become one of the cornerstones of the global energy transition, in line with the goals of combating climate change and ensuring security of energy supply. According to Global Wind Energy Council (GWEC) data, 165 GW of new wind capacity was commissioned worldwide in 2025 and cumulative installed capacity reached 1299 GW [1]. However, the stochastic, intermittent, and non-stationary nature of wind speed creates significant uncertainty for generation planning, grid stability, and imbalance costs in electricity markets [2,3]. Improvements in short-term wind speed forecast accuracy are therefore critical for the safe and economic integration of wind energy into the grid.

Four main approaches are used in the literature for wind speed prediction: physical (numerical weather prediction) models, classical statistical methods (e.g., the ARIMA family), machine learning-based methods and deep-learning architectures [3,4]. Although classical statistical models represent linear dependencies well, they cannot capture the non-linear and non-stationary dynamics of wind speed [4]. In order to overcome this limitation, the decomposition–prediction–integration paradigm, which is based on decomposing the signal into more stationary sub-components and modeling each component separately, has become the dominant framework in the field in recent years [2,5,6]. In this paradigm, the raw wind speed series is decomposed into sub-bands using methods such as discrete wavelet transform (DWT) [7] or variational mode decomposition (VMD). The sub-bands are then forecast with sequence models such as Bidirectional Long Short-Term Memory (BiLSTM) [8] and the final prediction is obtained by combining the component predictions [5,6,9].

The performance of hybrid decomposition–deep-learning models strongly depends not only on the model architecture, but also on decomposition parameters such as the mother wavelet type and the decomposition level and architectural and training hyperparameters such as look-back window length, network size, and learning rate [3,10]. In these high-dimensional search spaces, which contain categorical and continuous variables together, manual tuning and grid search are inadequate in terms of both computational cost and objectivity. Therefore, Bayesian optimization techniques [3] and population-based metaheuristic algorithms [10,11] have been widely adopted for hyperparameter optimization.

However, three important gaps stand out in the existing literature. First, the majority of wind forecasting studies propose a single metaheuristic algorithm. Systematic studies comparing the relative performance of different algorithms on the same problem with equal computational budget and sufficient number of iterations are quite limited. Existing metaheuristic comparisons in time-series forecasting have either been conducted in non-wind domains without decomposition components [12], focused on feature selection rather than hyperparameter optimization [13], or restricted to a small number of algorithms without joint search across the full architecture [11]. Second, decomposition parameters and model hyperparameters are typically fixed either through preliminary experiments [4,6,9] or are optimized sequentially in two separate stages [14,15]. Joint optimization, which can capture the interactions between the two sets of parameters, is rarely addressed, and the superiority of the joint approach over the sequential approach has not been quantitatively demonstrated through controlled experiments to date. Third, the performance of the new-generation metaheuristics proposed in 2025–2026 on real-world problems, and particularly on wind forecasting, has not yet been tested in independent studies.

Motivated by these gaps, our study addresses four research questions in the context of DWT–BiLSTM-based short-term wind speed prediction: (RQ1) which metaheuristic algorithm produces lower and more stable errors in the nine-dimensional combined search space covering decomposition, architecture and training hyperparameters? (RQ2) Does joint optimization provide measurable gains compared to the sequential approach commonly adopted in the literature? (RQ3) Can next-generation metaheuristics compete with established algorithms? (RQ4) Does hyperparameter search conducted on a single-year data subset produce robust results on multi-year data?

We frame architectural design as a unified search problem: rather than fixing decomposition parameters a priori from domain expertise, we let population-based bio-inspired metaheuristics jointly optimize nine hyperparameters spanning the signal-processing front-end (wavelet, decomposition level, boundary mode) and the recurrent backbone (look-back, hidden size, layers, dropout, learning rate, batch). The methodological contribution is threefold: (i) reformulating the conventional sequential, expert-driven tuning workflow as a single search problem, (ii) systematically quantifying how the choice of bio-inspired search strategy shapes the discovered architectures, and (iii) attributing predictive contribution to individual architectural components via ablation. The main contributions of the study can be summarized as follows:

- Eight metaheuristic algorithms are compared for wind speed prediction (Artificial Bee Colony (ABC) [16], genetic algorithm (GA) [17], Particle Swarm Optimization (PSO) [18], Grey Wolf Optimizer (GWO) [19], Hippopotamus Optimization (HO) [20], Farthest-better Nearest-worse Optimizer (FNO) [21], Raindrop Optimizer [22], Tuckman Optimization Algorithm (TOA) [23]) under a common experimental protocol with an equal computational budget and 30 independent runs, together with statistical significance tests and effect size measures.
- An optimization framework is proposed that combines decomposition (mother wavelet, level, fill mode), architecture (look-back window, number of hidden units, number of layers, dropout) and training (learning rate, batch size) parameters into a single nine-dimensional search space, enabling a direct experimental comparison of the joint and sequential paradigms.
- The FNO, Raindrop and TOA methods are evaluated for the first time in wind speed forecasting with decomposition-integrated deep-learning models, providing an application reference point for these algorithms.
- The robustness of the one-year search is examined through a multi-window analysis and through a genuine rolling-origin out-of-sample evaluation with a strict nested train/validation/test protocol, alongside component and wavelet-family ablation analyses.
- Beyond the algorithm comparison, a further contribution of this work is methodological. Decomposition-based deep-learning forecasters are frequently evaluated in ways that can flatter them, for example, by decomposing the full series before the train/test split, or by reporting errors in the normalized or per-component space rather than for the reconstructed signal. We adopt a leakage-controlled, physically meaningful protocol: the wavelet decomposition is applied causally, forecasts are reconstructed to physical units (m/s), and every configuration is compared against a naive persistence baseline on an untouched test set. This protocol yields an honest and instructive picture of what the architecture does and does not achieve at the one-hour horizon, and we report it transparently.

The remainder of the work is organized as follows. Section 2 examines the relevant literature under four subsections and clarifies the literature gap. Section 3 introduces the dataset, the DWT–BiLSTM architecture, and the common experimental protocol to which the eight algorithms were subjected. Section 4 presents the experimental results in four stages: joint optimization benchmark, joint–sequential comparison, multi-window robustness and rolling-origin evaluation, and ablation analyses. Section 5 discusses the findings in the context of the existing literature; Section 6 summarizes the results and limitations of the study and future study directions.

## 2. Related Works

This section positions the present study within the existing literature and substantiates, through concrete studies, the gaps identified in Section 1.

### 2.1. Decomposition-Based Hybrid Deep-Learning Models

Numerous hybrid frameworks combining decomposition methods and deep-learning models have been proposed to address the non-stationary nature of the wind speed signal. Barjasteh et al. modeled the DWT-decomposed signal with BiLSTM and BiGRU architectures and reported a 16–26% improvement in RMSE over the baseline methods, but determined the model hyperparameters manually [5]. Li et al. fed the VMD-decomposed series into a PSO-optimized BiLSTM model for offshore typhoon wind speed forecasting. However, the VMD parameters (number of modes K and penalty coefficient) were fixed by preliminary experiments [6]. In the GA-VMD–SE–BiLSTM model proposed by Liu and Liu, GA optimizes only the VMD parameters, while the BiLSTM hyperparameters remain constant [15]. Similarly, Zhang et al. used the Dung Beetle Optimizer (DBO) algorithm only for the selection of VMD parameters, keeping the structure of the TCN–Transformer prediction block constant [9]. Xiong et al. presented a dual-scale model in which an improved reptile search algorithm (IRSA) optimizes BiLSTM and ELM models within a preprocessing pipeline based on sample entropy and random forests; here too, the decomposition side remained outside the scope of optimization [24]. Gu et al. applied the wind power series decomposed by SVMD to the TCN–BiGRU model whose hyperparameters were adjusted with the RIME algorithm [25].

Even the studies that come closest to considering decomposition and model parameters together follow a sequential structure. Within the dual-optimization framework proposed by Li et al., the genetic algorithm (GA) optimizes the VMD parameters, while the improved dung beetle optimization (IDBO) optimizes the hyperparameters of the BiLSTM-Attention model in separate stages [14]. Amirteimoury et al. performed mutual information (MI)-based input selection on the DWT-decomposed signal using the Coot optimization algorithm, but the optimization was limited to a single scalar threshold and the BiLSTM hyperparameters were used as predefined constant values [4]. In summary, in all studies in this cluster, decomposition and model parameters are either partially fixed or considered sequentially in separate search spaces. Possible interactions between the two parameter layers are therefore excluded from the search process.

### 2.2. Metaheuristic Hyperparameter Optimization for BiLSTM

The use of metaheuristic algorithms in hyperparameter tuning of BiLSTM architecture has become widespread in recent years. Alhussan et al. tuned BiLSTM parameters with the hybrid GADTO algorithm, which combines the genetic algorithm with Dipper Throated Optimization, and supported the significance of the results with ANOVA and Wilcoxon signed-rank tests [26]. The same research group proposed a hybrid ensemble model based on whale optimization for wind power forecasting [27]. Yousef et al. presented LSTM/GRU-based hybrid architectures tuned with the Snake Optimizer along with explainable artificial intelligence (XAI) analysis [28]. Lee et al. applied improved artificial protozoa optimization (IAPO) to an LSTM-based prediction framework [29]. Huang et al. proposed the EvLSTM model, which co-evolves the LSTM architecture and hyperparameters with the genetic algorithm [30]. This approach comes close to joint optimization in that it handles the model structure and training parameters within a single process, but it does not include signal decomposition. A common feature of the studies in this cluster is that they employ a single algorithm, usually newly proposed or improved, and do not present a systematic comparison between algorithms.

### 2.3. Multi-Algorithm Benchmarks and Optimization Paradigms

Multi-algorithm comparison studies are relatively few and have mostly been conducted in areas other than wind speed forecasting. Sen et al. compared GA, differential evolution (DE) and PSO algorithms on ANN, LSTM, GRU and ARIMA models in the context of temperature prediction, limiting the search space to three hyperparameters and reporting the average of five runs for each algorithm [11]. The authors’ finding that there is no previous study that comprehensively compares these three basic evolutionary algorithms across more than one architecture is noteworthy, as it illustrates the limitations of the comparative literature in this field. El-kenawy et al. compared six metaheuristics in the context of wind speed prediction, but this comparison was made for the purpose of feature selection, not hyperparameter optimization [13]. Alharbi et al. proposed the iHow Optimization Algorithm (iHowOA), a human-cognition-inspired metaheuristic, for BiLSTM hyperparameter tuning, benchmarking it against nine competing algorithms on an IoT-based HVAC energy consumption dataset [12]. However, its application area is HVAC energy consumption forecasting, it does not include signal decomposition, and the search space is limited to BiLSTM hyperparameters. The systematic review by Mumtahina et al. on load forecasting shows that metaheuristic hyperparameter tuning has become common in adjacent energy forecasting problems, whereas including the decomposition layer within the scope of optimization remains exceptional [10].

Bayesian optimization frameworks are also used for hyperparameter tuning alongside the metaheuristic paradigm. Hanifi et al. compared Optuna, Hyperopt and Scikit-opt frameworks for hyperparameter optimization of CNN and LSTM models; TPE-based Optuna stood out in terms of efficiency [3]. In the current study, eight population-based metaheuristic algorithms are compared under equal conditions on the same DWT–BiLSTM problem.

Beyond population-based metaheuristics, exact and relaxation-based mathematical-programming methods offer an alternative optimization paradigm for renewable-energy systems; for instance, semi-convex relaxation has been applied to hybrid-microgrid scheduling with hydrogen storage under data-correlated uncertainty [31]. Such formulations trade broad applicability for problem-specific optimality guarantees, complementing the derivative-free, black-box search studied here.

### 2.4. Literature Gap and Position of This Study

Table 1 summarizes the representative studies reviewed in terms of application area, use of decomposition, scope of optimization, number of algorithms, number of iterations and statistical testing. Three gaps are clearly observed from the table: (i) in the field of wind speed prediction, there is no study comparing five or more metaheuristic algorithms under a common protocol for hyperparameter optimization. (ii) There is no study that combines the decomposition parameters with the model and training hyperparameters in the same search space and compares this combined setup with controlled experiments with sequential editing. (iii) Wind forecasting application of FNO, Raindrop and TOA methods have not been reported yet. This study addresses these three gaps together by combining an eight-algorithm iterative benchmark, nine-dimensional joint optimization, and paired joint–sequence experiments in a single framework.

## 3. Materials and Methods

This section introduces the dataset and preprocessing steps, the DWT-based signal decomposition and the BiLSTM prediction architecture, and then it presents the nine-dimensional joint hyperparameter optimization problem. It also describes the eight compared metaheuristic algorithms, the four-stage experimental protocol, and the evaluation criteria and statistical analysis methods.

### 3.1. Dataset and Preprocessing

Hourly wind speed measurements from eight meteorological stations in the Nevşehir province of Türkiye were used. Each station provides 51,144 continuous hourly observations (approximately 5.84 years). Across the dataset, the mean wind speed is approximately 1.97 m/s with a standard deviation of 1.45 m/s, and measurements range from 0 to 10.2 m/s. The relatively low mean speed and high variability characteristic of central Anatolia provide a demanding test environment for the forecasting problem.

In order to preserve the integrity of the time series, the data were divided chronologically without shuffling, with the first 80% of each signal reserved for training and the remaining 20% for validation and testing. Min–max normalization was applied to eliminate scale sensitivity. In order to prevent data leakage, the scaler was fitted only on the training partition, and the same transformation was applied to the test partition. The hyperparameter search was conducted on the final 8760 h (one-year) slice of each station in order to keep the computational cost manageable. The robustness of this design choice is tested further through the multi-window robustness and rolling-origin evaluation experiments reported in Section 4 (RQ4). The final training of the best configurations was carried out on all 51,144 h of data. Concretely, for the benchmark comparison the first 40,915 hourly observations (≈80%) of each station form the training partition and the final 10,229 (≈20%) the test partition; because this split alone does not isolate the test period from hyperparameter selection, the strict out-of-sample assessment additionally uses a nested train/validation/test partition organized by whole years (rolling-origin protocol, Section 3.6).

### 3.2. Signal Decomposition with Discrete Wavelet Transform

The discrete wavelet transform (DWT) decomposes a signal into different frequency bands within the framework of multi-resolution analysis and can be computed efficiently with Mallat’s pyramid algorithm [7]. In an L-level decomposition, the signal is separated into L detail components ($D_{1},\ldots ,D_{L}$) and one approximation component ($A_{L}$). The approximation component represents the low-frequency trend, and the detail components represent oscillations in increasingly higher-frequency bands. The nature of the decomposition depends on three design choices: the mother wavelet function, the decomposition level L, and the padding mode at the signal ends. In this study, a total of 12 mother wavelet candidates from Daubechies (db4–db8), Symlet (sym5–sym8) and Coiflet (coif3–coif5) families [32], with levels in the range 3–7 and three fill modes (symmetric, zero, periodization), are included in the search space. Contrary to common practice in the literature, these three parameters are not fixed by preliminary experiments, but are optimized as components of the joint search space defined in Section 3.4. DWT calculations were performed with the PyWavelets 1.8.0 library [33].

To avoid look-ahead information leakage from the boundary and periodization operators of the wavelet filters, the decomposition is applied causally: the series is first partitioned chronologically, and each partition (training, validation and test) is decomposed separately, so that no future observation can influence the wavelet coefficients used for training. A coefficient-level comparison (Section 4.5) shows that this causal scheme and the original full-series decomposition leave more than 99.9% of the training coefficients identical; the correction therefore does not materially change the reported rankings, while removing the leakage in principle.

### 3.3. BiLSTM Prediction Architecture

Long short-term memory (LSTM) networks are recurrent neural networks that learn long-range dependencies through gating mechanisms and alleviate the vanishing gradient problem [34]. The bidirectional variant (BiLSTM) processes the input sequence with two separate forward and backward LSTM layers, representing past and future context together at each time step [8]. In this study, a separate BiLSTM model is trained for each DWT component. The model for component *b* takes the most recent *w*-hour window (look-back, $w\in \left[24,96\right]$) as input and forecasts one hour ahead (H=1). After inverse normalization, the component forecasts are recombined with the inverse DWT to obtain the final wind speed forecast in physical units (m/s). The models were trained with the Adam optimizer and a mean squared error (MSE) loss, with gradient-norm clipping (maximum 1.0) to prevent exploding gradients and early stopping to limit overfitting. All models were implemented with PyTorch 2.9.1 (CUDA 12.6 build).

### 3.4. Formulation of the Joint Hyperparameter Optimization Problem

**Figure 1:**
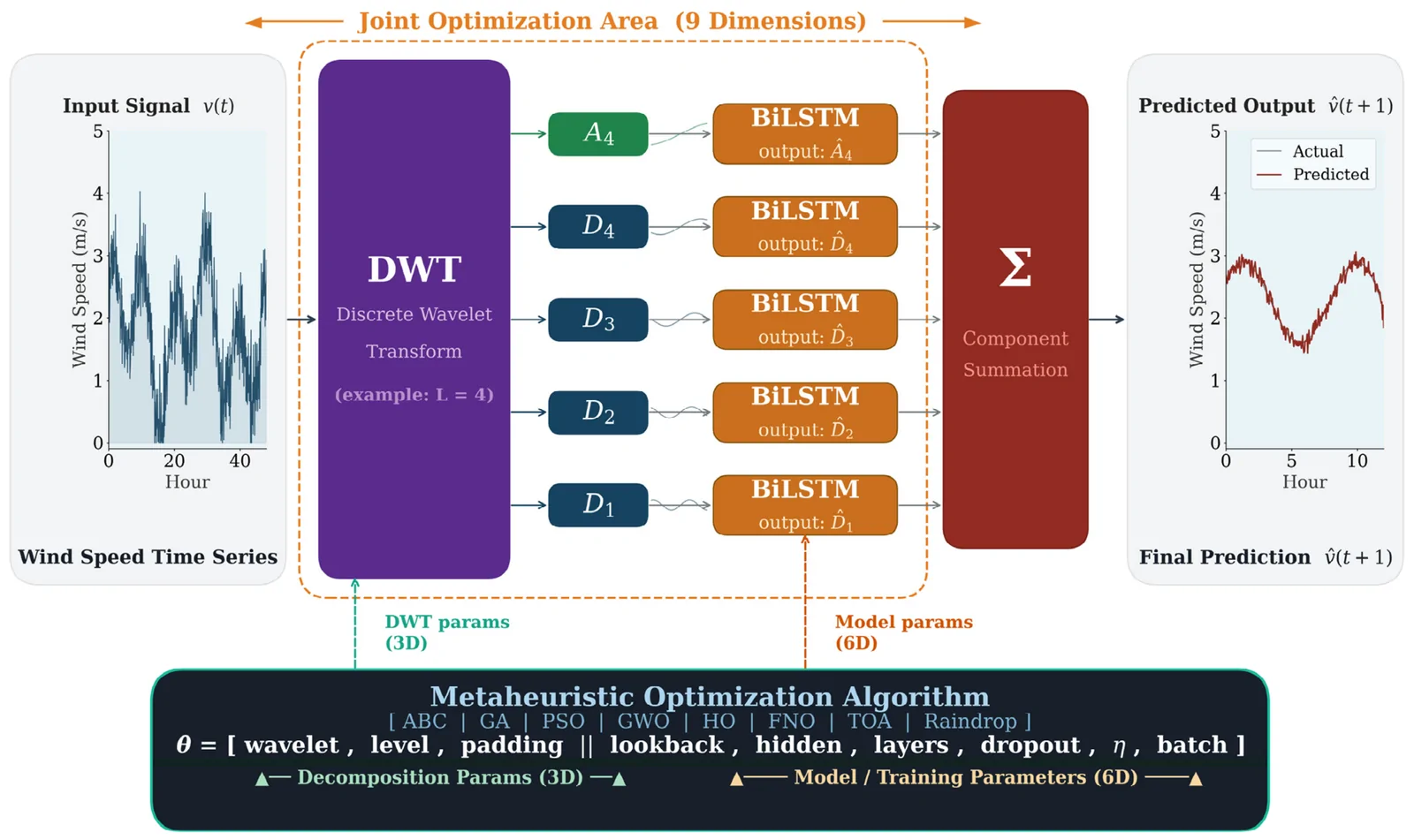
DWT–BiLSTM joint hyperparameter optimization system architecture. The decomposition (3D) and architecture/training (6D) parameters are optimized jointly in a single nine-dimensional search space [population = 40; iterations = 50; 2000 evaluations per run]. Solid arrows indicate the forward data flow, whereas dashed arrows indicate the hyperparameters supplied by the metaheuristic optimizer (green: decomposition parameters; orange: model and training parameters). The orange dashed frame encloses the parameters that are optimized jointly.

In the proposed framework, decomposition, architecture and training parameters are combined in a single search space. Figure 1 illustrates the joint optimization process. The decision vector has nine dimensions, θ = (wavelet, level, fill mode, w, hidden unit, layer, dropout, learning rate, batch size), and contains a combination of categorical, integer and continuous variables (see Table 2). To ensure that all algorithms see the same problem in this heterogeneous type of space, the search was carried out on the unit hypercube [0,1]^9^ and converted to real parameter values with a deterministic mapping. Categorical dimensions were divided into equal-width intervals, integer dimensions were rounded with inclusive edges, and the learning rate was mapped on a logarithmic scale. Ensuring that the same vector corresponds to the same configuration in every algorithm is a prerequisite for fair comparison, and reducing the mixed-type search space to a uniform representation allows the high-dimensional categorical–continuous interactions to be searched consistently.

The parameter ranges were not chosen arbitrarily. The wavelet families and level ranges cover values commonly used in the wind speed literature, together with the region that gave stable results in our preliminary experiments. The architecture and training ranges were set wide enough to span both very small models (underfitting) and very large ones (overfitting and high computational cost). The objective function is the average of validation RMSE values calculated on a component-by-component basis:

(1) $$f\left(\theta \right)=\frac{1}{L+1}\sum_{b\in A_{L},D_{1},\ldots ,D_{L}}RMSE_{val}\left(b\right)$$

During the search, each evaluation was conducted with an abbreviated training protocol (20 epochs, early stopping patience of 5) to limit the computational budget. The final models were trained with the full protocol (100 epochs, patience of 15). The aim of this surrogate evaluation strategy is to reduce the cost per run by a factor of approximately five while preserving the relative ordering of configurations during the search phase.

It should be emphasized that the objective in Equation (1), namely the equally weighted average of the normalized per-sub-band validation RMSE, is a search surrogate and is not equivalent to the forecasting error of the reconstructed wind speed signal. Selected configurations are therefore additionally validated in reconstructed physical units (m/s) after inverse DWT and inverse scaling (Section 3.7 and Section 4.5).

### 3.5. Metaheuristic Algorithms Compared

The comparison set spans the major families of bio-inspired computation. Four are well-established: the swarm-intelligence Artificial Bee Colony (ABC), which balances exploration and exploitation through worker–onlooker–scout bee roles [16]; the evolutionary genetic algorithm (GA), based on selection, crossover and mutation [17]; Particle Swarm Optimization (PSO), in which particles move toward individual and social optima [18]; and the Grey Wolf Optimizer (GWO), which models the pack hierarchy of alpha, beta and delta leaders during prey encirclement [19]. Four are recent: Hippopotamus Optimization (HO), a swarm method built on in-stream positioning, defense and avoidance phases [20]; the Farthest-better/Nearest-worse Optimizer (FNO), a deliberately metaphor-free, distance-based algorithm that orients each individual by farthest-better and nearest-worse reference points [21]; the nature-inspired Raindrop Optimizer, which turns raindrop splashing, gravitational flow, evaporation and overflow into search operators [22]; and the Tuckman Optimization Algorithm (TOA), a social-behavior method embedding Tuckman’s group development stages into population search [23]. This set deliberately contrasts classical animal- and evolution-inspired mechanisms with newer metaphor-free and social paradigms, enabling a direct test of whether biological metaphor confers a search advantage on this problem. The eight methods were chosen to represent the principal metaheuristic families in balanced proportion, four long-established and four proposed in 2024–2026, rather than to provide an exhaustive census; this keeps the comparison interpretable while still spanning swarm, evolutionary, leader–follower, metaphor-free and social-behavior paradigms.

For a fair comparison, all algorithms used the same [0,1]^9^ search space, the same parameter mapping and the same objective function, with the population size fixed at 40 and the maximum number of iterations at 50 (2000 objective-function evaluations per run). No algorithm-specific tuning was performed: every control parameter was kept at the default value recommended in the corresponding original publication, and this identical search budget was applied to all eight algorithms, with no adjustment made in favor of any method. This deliberately avoids favoring any algorithm through hand-tuning and isolates the effect of the search mechanism itself. The specific control-parameter values used for each algorithm are reported in Table A1 (Appendix A).

### 3.6. Experimental Protocol

The experiments were designed in four stages that map directly onto the four research questions in Section 1.

*Stage 1 (benchmark, RQ1 and RQ3)*: Each of the eight algorithms performed 30 independent runs with different random seeds. Because single-run reporting can be misleading given the stochastic nature of metaheuristics, a distribution-based comparison is used.

*Stage 2 (joint–sequential comparison, RQ2)*: In the sequential setup, the decomposition parameters were selected and fixed by the Shannon entropy criterion in the first stage, and in the second stage, the remaining six dimensions were optimized with the same algorithm and the same total budget. The joint and sequential setups were compared run by run, paired through the same seed set.

*Stage 3 (multi-window robustness analysis, RQ4)*: The data were divided into five consecutive annual windows (the initial 10-month segment was excluded because it did not constitute a full year) and the hyperparameter search was repeated independently in each window. The best configuration from each window was then retrained on the entire dataset, and the sensitivity of the single-year search strategy to window selection was measured.

*Stage 4 (ablation)*: Two ablation scenarios, in which the DWT layer was removed (the raw signal was fed directly to BiLSTM) and bidirectionality was turned off (unidirectional LSTM), were run using the same seeds as Stage 1, and the contribution of each component was quantified in paired tests.

The computing infrastructure is two-tiered: hyperparameter searches were run with 40 parallel workers on 40-core processor nodes of the national academic computing resource TRUBA. Final model training was carried out on a local GPU with CUDA support. The total experimental load corresponds to training 8 algorithms × 30 runs × 2000 evaluations = 480,000 surrogate models for Stage 1 alone. For reproducibility, all run configurations, seed values and per-iteration trial histories are stored in SQLite databases. The complete code base, together with the trial-history databases and aggregated result JSONs that allow verification of every published number, figure, and table, is openly archived at Zenodo (https://zenodo.org/records/21384094) [35] and mirrored at GitHub (https://github.com/emrebendes/wind-dwt-bilstm-metaheuristic, accessed on 4 August 2026). The raw wind speed time series are subject to the data-sharing terms of the Turkish State Meteorological Service (MGM) and are not redistributed; station-level summaries are included in the Zenodo deposit.

To support a genuinely out-of-sample assessment, a strict nested protocol is additionally adopted: in the rolling-origin evaluation each origin is trained only on past years, early-stopped on the subsequent year, and evaluated on a later, completely untouched test year that enters neither hyperparameter selection nor training. Each selected configuration is retrained under multiple neural-network seeds so that training-induced variance is estimated separately from metaheuristic-induced variance. Because the earlier Stage 3 retrains on the full series, it is reported as a multi-window robustness analysis rather than as walk-forward validation (Section 4.3).

### 3.7. Evaluation Criteria and Statistical Analysis

Forecast performance is reported in terms of RMSE, MAE and the coefficient of determination (R^2^). For optimization performance, the best objective function value (validation RMSE) of each run is used. The algorithms were compared over their 30-run distributions following the non-parametric framework recommended for multi-method comparisons [36]. First, the Friedman test [37] was used to determine whether there was a general difference among the algorithms. When a significant difference was found, pairwise comparisons were performed using the Wilcoxon signed-rank test [38], and the inflation of Type I errors due to multiple comparisons was controlled using the Holm correction [39]. To evaluate statistical significance in conjunction with practical significance, the effect size measures Cohen’s d [40] and the rank-based Cliff delta [41] were reported. During the multi-window robustness analysis stage, differences between windows were analyzed using the Kruskal–Wallis test [42] and subsequent Mann–Whitney U tests [43] (with Holm correction) for independent samples, while the joint–sequential and ablation comparisons were analyzed with the paired Wilcoxon test, since the use of identical seeds provides a paired design. In all tests, the significance level was taken as 0.05.

In addition to the normalized search metric, forecasts are evaluated in reconstructed physical units: after inverse DWT and inverse scaling, station-level RMSE, MAE and R^2^ are reported in m/s, together with range- and mean-normalized RMSE and, for observations above a small wind speed threshold, MAPE (which is unstable near zero and is reported with this caveat). Uncertainty is quantified with 95% bootstrap confidence intervals, and all reconstructed-space forecasts are compared against the naive persistence baseline of Section 3.8.

### 3.8. Naive Persistence Baseline

To establish a lower-bound reference and to quantify the value added by the learned model beyond serial correlation, a naive 1-step persistence forecast is computed as $\hat{y}\left[t\right]=y\left[t-1\right]$. This zero-learning predictor reflects the predictability arising solely from the autocorrelation of wind speed and is the recommended minimum reference baseline in operational wind forecasting [44]. The skill score with respect to persistence is defined as:

(2) $$SS=1-\left(RMSE_{model}/RMSE_{persistence}\right)$$

where *SS* > 0 indicates that the learned model provides predictive value beyond persistence [45]. Persistence RMSE is computed on the same chronological 20% test partition used for model evaluation. To make the skill score directly comparable with the surrogate metric reported throughout this study, persistence RMSE is computed both on the raw wind speed series (in m/s) and on each normalized DWT component, with the mean across stations and components serving as the reference value.

## 4. Experimental Results

This section reports the results of the four-stage experimental protocol described in Section 3.6. All statistical calculations were performed with SciPy 1.15.2 (Python 3.10.19) and the statistical significance level was adopted as α = 0.05. To assess practical significance, parametric effect size (Cohen’s d) and rank-based effect size (Cliff δ) measures are reported together.

### 4.1. Joint Optimization Results

In the first stage, each of the eight metaheuristic algorithms described in Section 3.5 was run 30 times independently, with different random seeds, in the nine-dimensional joint search space. Each run performed 2000 objective function evaluations with a population size of 40 and a maximum of 50 iterations. The best validation RMSE of each run was used as the key metric for comparison between algorithms. Table 3 presents the run-by-run distribution statistics in order of mean RMSE. The final RMSE column reports the result of the final training performed on the entire 51,144 h dataset with the configuration of each algorithm’s best run.

In Table 3, the Rank column is the average taken over 30 seeds after ranking the algorithms according to RMSE (1= best, 8 = worst) for each seed within the framework of the Friedman test. The lower rank indicates a tendency to lead, or to be close to the leader, across multiple seeds. The Friedman test shows a statistically significant difference between the median performances of the eight algorithms (χ^2^ = 49.76; degrees of freedom = 7; *p* < 10^−8^; *n* = 30). The average rank (ĀR) is a more robust indicator of leadership than the run-based mean, because it summarizes the rank distribution of an algorithm across 30 separate seeds. According to the average rankings, the top three algorithms are GWO (2.27), PSO (2.97) and GA (4.00). This ranking is the main justification for choosing GWO as the reference algorithm in the later stages of the study (Stages 2, 3 and 4): GWO not only attains a low average RMSE but also shows reliable leadership, ranking in the top two positions in nearly three-quarters of the 30 seeds. GWO leads in all run-based metrics, with the lowest mean (0.0371), the lowest median (0.0366) and the lowest dispersion (standard deviation 0.00199, corresponding to a coefficient of variation of only 5.37%). GA stands out in the single-run minimum column (0.0340); however, its mean of 0.0392 indicates that this result reflects a “lucky run”.

**Figure 2:**
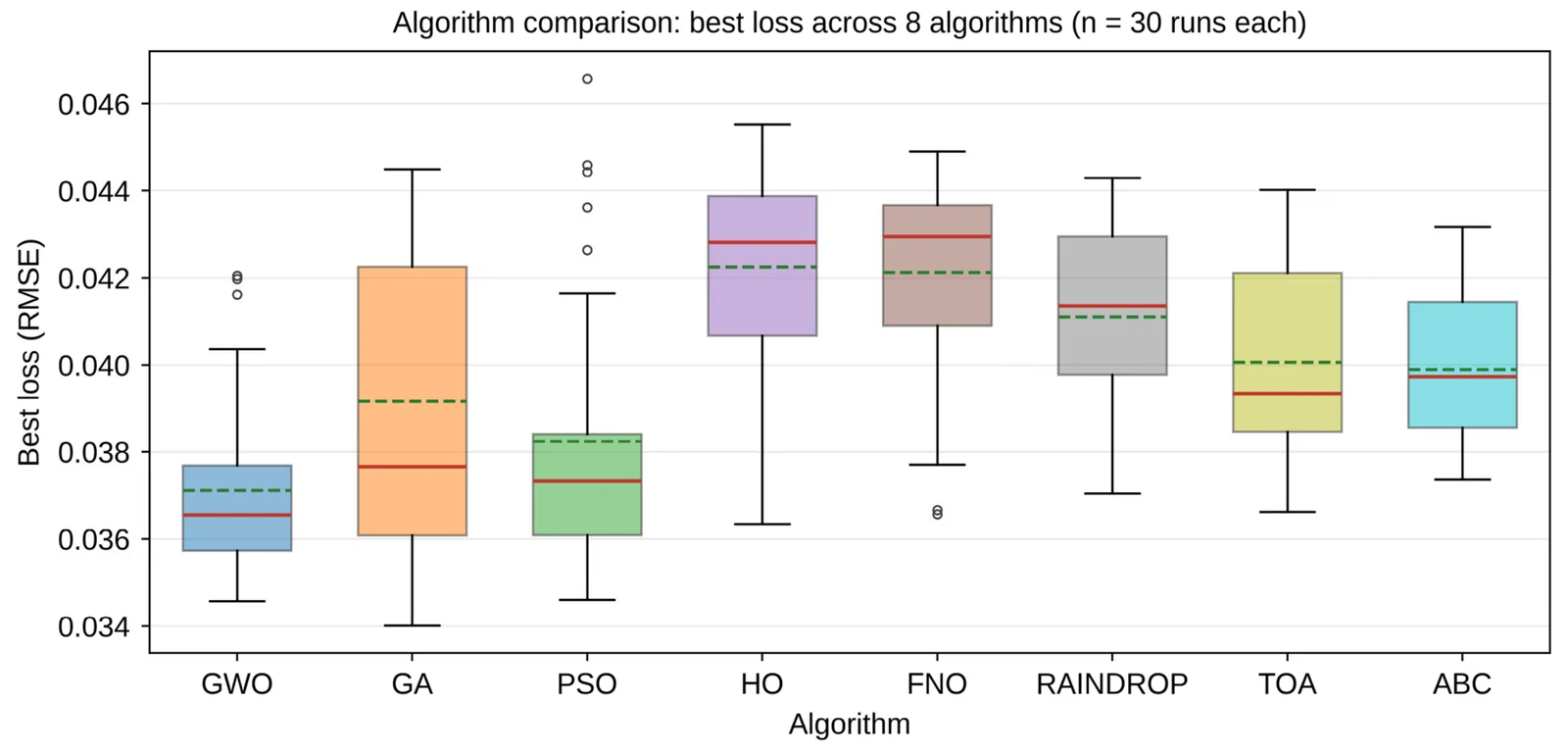
A comparison of the results of 8 meta-heuristic optimization algorithms across 30 runs. In each box, the red solid line is the median, the green dashed line is the mean, the box spans the interquartile range (IQR), the whiskers extend to 1.5× IQR, and open circles are outliers. Colours distinguish the algorithms and carry no additional meaning.

The new-generation algorithms (TOA, Raindrop and FNO) underperformed the classical metaheuristics in terms of average RMSE. This finding constitutes a partially negative answer to RQ3. However, the final training RMSEs of the algorithms are quite close to those of the classical ones (range 0.0782–0.0793). The Raindrop algorithm achieved the lowest final RMSE, 0.07819. The RMSE values obtained by the eight algorithms are shown in Figure 2.

The GWO configuration obtained in the best run of the winner of the first stage is as follows: main wavelet = db4, decomposition level = 4, fill mode = symmetric, look-back = 91 h, number of hidden units = 85, number of layers = 1, dropout = 0.432, learning rate = 10^−2^ and batch size = 32. This configuration is used as a reference in the subsequent stages.

**Figure 3:**
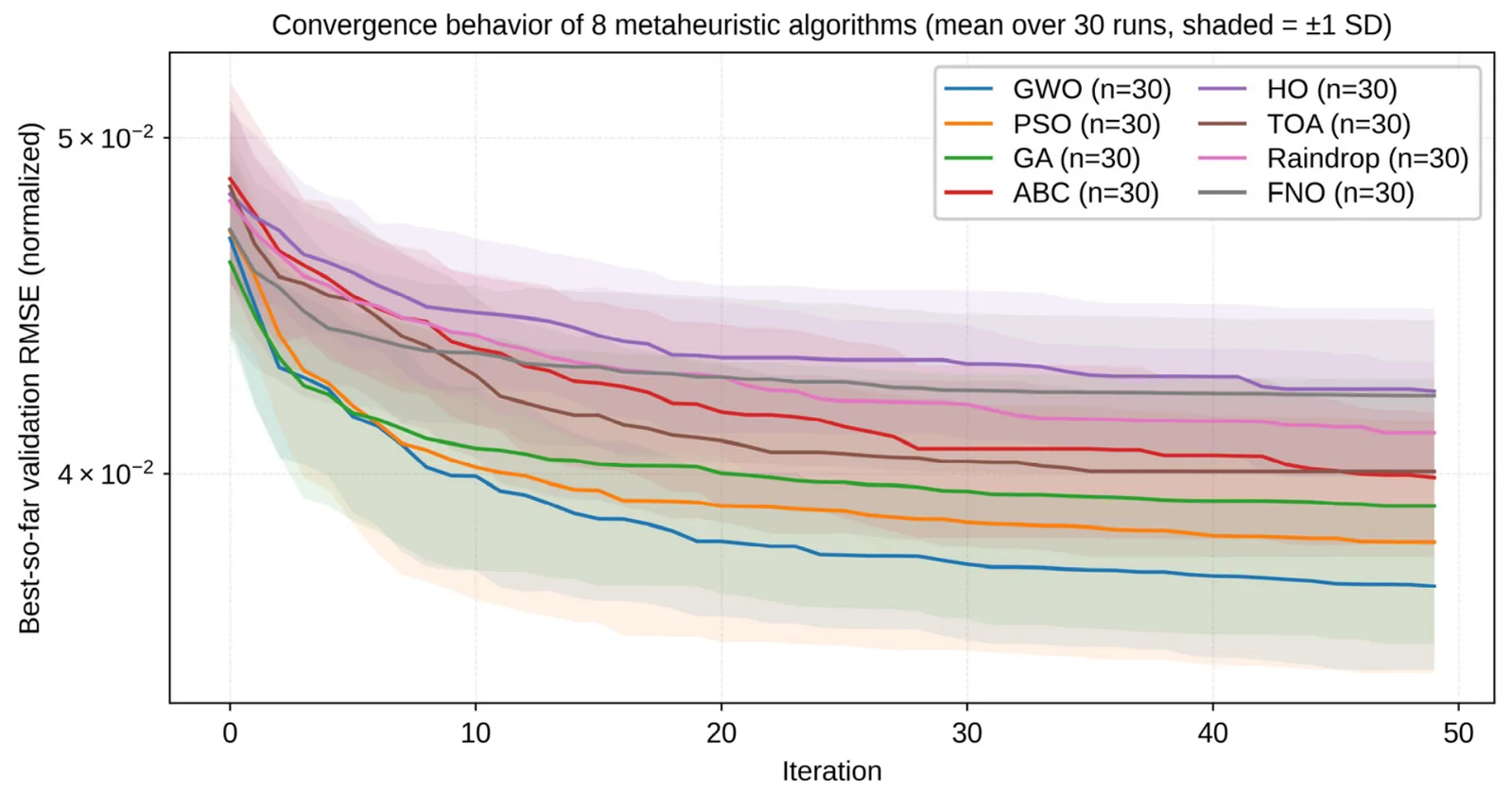
Convergence behavior of the eight metaheuristic algorithms (mean of 30 independent runs, shaded ±1 SD). The y-axis (best-so-far validation RMSE) is in logarithmic scale. GWO consistently achieves the lowest plateau.

To complement the run-level statistics in Table 3, the best-ever validation RMSE per iteration was tracked for each algorithm. Figure 3 shows the average RMSE and standard deviation for all eight algorithms over 50 iterations, based on 30 runs. GWO, PSO, and GA enter the lowest plateau within the top ten iterations, while HO, FNO, and Raindrop remain at a higher plateau. ABC plateaus at approximately 0.040, above the GA/PSO cluster but below HO, FNO, and Raindrop. The relative ranking of the eight algorithms based on their average best loss value is consistent with the run-level Friedman ranking reported in Table 3, with GWO consistently dominant from approximately 15 iterations onwards.

**Figure 4:**
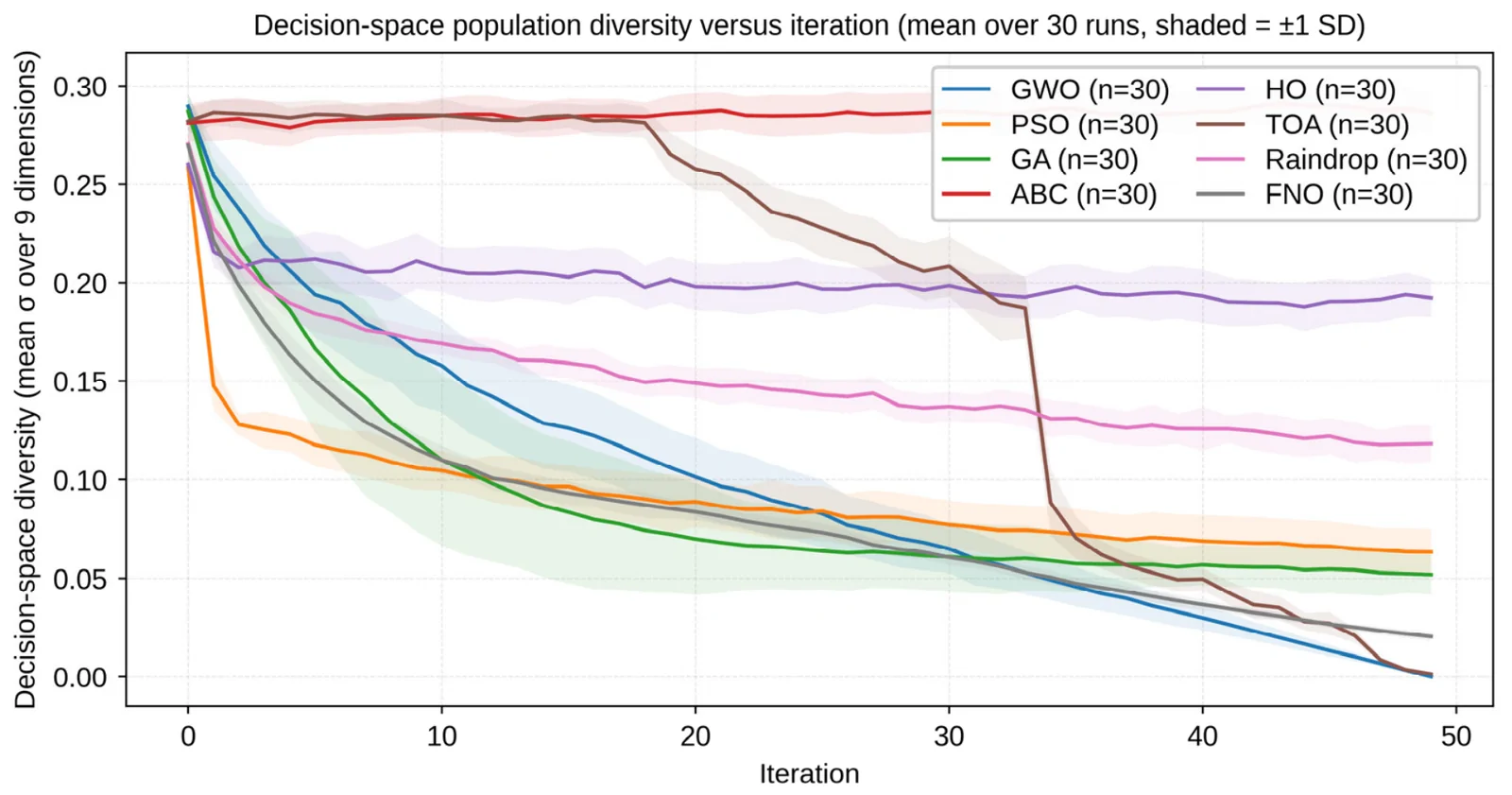
Decision-space population diversity (mean σ over 9 normalized dimensions) versus iteration, for each algorithm (30-run mean). High diversity denotes exploration; low diversity denotes exploitation. ABC fails to collapse, while GWO and TOA achieve complete exploitation.

Beyond the fitness trajectory, the decision-space behavior of each algorithm is informative about its exploration–exploitation balance [46]. Figure 4 shows population diversity, measured as the mean of the per-dimension standard deviation across the min–max-normalized 9-D decision space, plotted against the iteration index. In other words, the figure measures how closely the 40 individuals of an algorithm cluster in the nine-dimensional space over time. High diversity indicates that individuals remain spread out. That is, the algorithm is in the exploration phase, whereas low diversity indicates the exploitation phase, in which individuals cluster around a single point that is then searched in detail. The decay ratio, calculated as the ratio of the final and initial diversity, indicates effective exploitation when it is close to 0 and a lack of convergence when it is close to 1.

GWO and TOA collapse to near-zero diversity (decay ratio < 0.01), reflecting strong exploitation. GA, PSO and FNO settle into intermediate diversity (decay ratio ≈ 0.08–0.25), retaining a small reservoir of exploration. Raindrop and HO plateau at decay ratios of 0.44 and 0.74 respectively, indicating incomplete refinement. Strikingly, ABC’s diversity ratio at the final iteration exceeds its initial value (1.018), a consequence of its scout phase periodically reseeding individuals at search-space corners. This mechanistic feature limits the late-stage exploitation from which GWO benefits, which is consistent with ABC’s fourth-place ranking despite an identical evaluation budget.

The wall-time cost of each algorithm is reported in Table 4. Mean total time per run is computed by summing iteration durations across all 30 runs and reporting the per-run mean and standard deviation. FNO is the fastest algorithm; GWO is the second-fastest (cost ratio 1.09× of FNO) while also achieving the lowest mean RMSE, making it Pareto-dominant on this benchmark. At the other extreme, ABC consumes 3.67× the wall-time of the fastest algorithm without competitive accuracy, making it a strict non-recommendation for this problem class.

Two consistency checks support the search protocol. First, re-evaluating all eight configurations with the causal (leakage-free) decomposition leaves the ordering essentially unchanged: the leaky and causal rankings correlate at Spearman ρ = 0.93 (*p* < 0.001), with a maximum change of 2.7% in any configuration, confirming that the boundary leakage does not affect the comparison. Second, on a common all-station, full-record setup the 20-epoch surrogate preserves the 100-epoch ranking at Spearman ρ = 0.74 (*p* = 0.04); the strongly negative correlation between the search stage and final scores noted informally in the original manuscript is an artifact of the differing data scope, not of the epoch count.

### 4.2. Joint–Sequential Optimization Benchmark

To test whether joint optimization is genuinely superior, the three best algorithms of the first stage (GWO, PSO and GA) were re-run on the same problem with a sequential optimization setup in the second stage. In the sequential setup, the decomposition parameters were first determined deterministically using the Shannon entropy criterion, after which the remaining six BiLSTM hyperparameters were optimized with the same algorithm under the same total budget (2000 evaluations) as in the joint setup. For a fair benchmark, the joint and sequential runs share the seed set of the first stage, which makes the paired Wilcoxon signed-rank test applicable.

**Figure 5:**
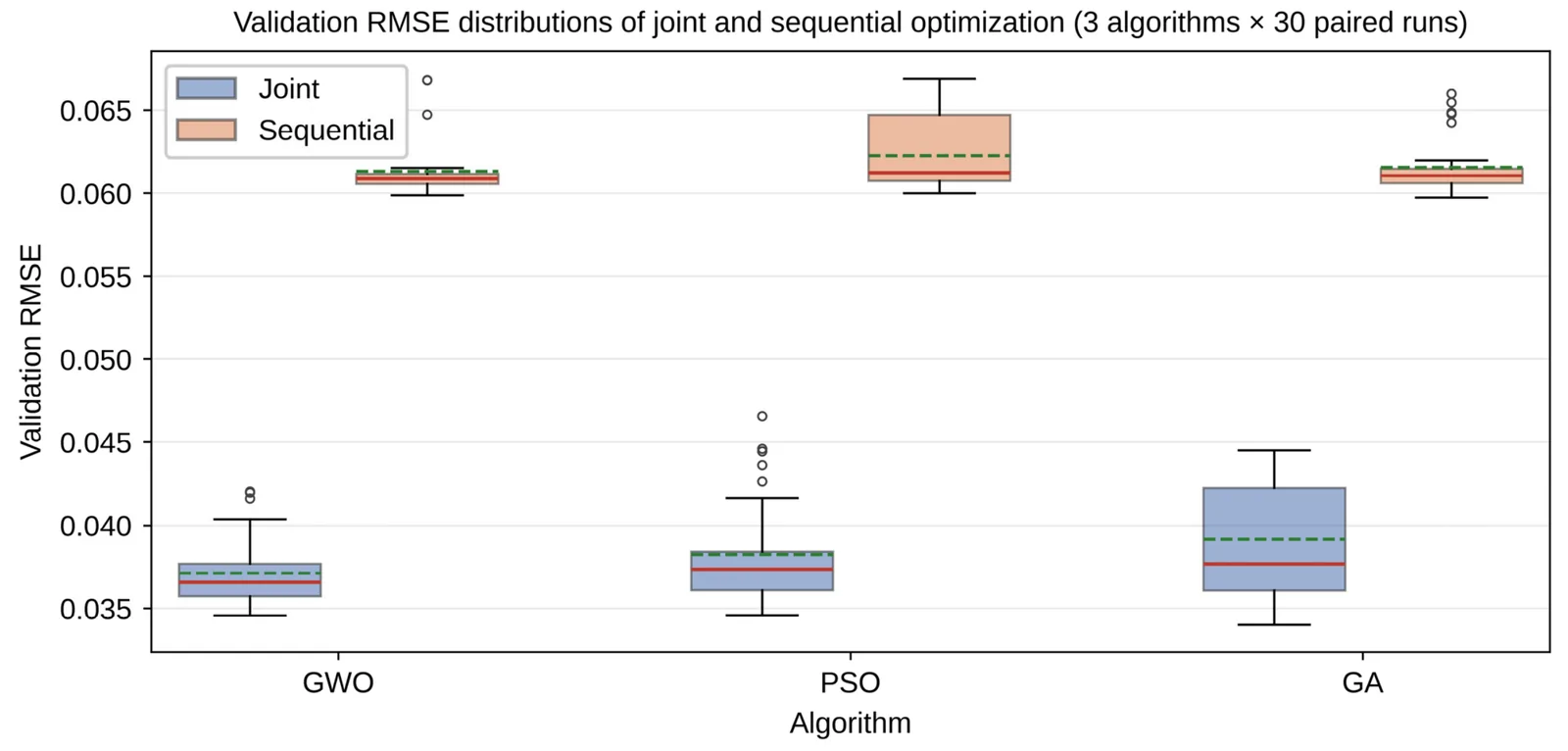
Validation RMSE distribution of joint and sequential optimization (3 algorithms x 30 paired runs). For each algorithm, left box: joint, right box: sequential optimization. In each box, the red solid line is the median, the green dashed line is the mean, the box spans the interquartile range (IQR), the whiskers extend to 1.5× IQR, and open circles are outliers.

The results in Table 5 show that joint optimization makes a substantial contribution. Across all 30 seed-matched run pairs for each of the three algorithms, joint optimization produced a lower RMSE than the sequential setup. Paired Wilcoxon test *p*-values remained below 10^−9^ for all three algorithms, and full ordinal dominance was achieved with Cliff δ = 1.00. In terms of practical magnitude, the median RMSE of the joint setup is approximately 39% lower than that of the sequential setup. This result indicates that selecting the decomposition parameters independently of the BiLSTM architectural decisions leads to suboptimal configurations. Crucially, this improvement is obtained at no additional search cost: joint and sequential optimization were run under the identical budget of 2000 objective evaluations, and each evaluation is dominated by the same BiLSTM training step in both schemes; joint search therefore reallocates the same budget across a coupled nine-dimensional space rather than adding computational overhead, so no accuracy–cost trade-off arises between the two paradigms. The RMSE distributions of the 30 paired runs for each algorithm are shown in Figure 5.

### 4.3. Multi-Window Robustness and Genuine Rolling-Origin Evaluation

To test the robustness of the single-year search strategy, the GWO algorithm, the winner of the first stage, was independently re-run in five separate annual windows (Y2–Y6, 8760 h each). Window Y1 is excluded from the window set because it only contains 7344 h (approximately 10 months). However, the final training for all windows was conducted on the full 51,144 h of data, so information from Y1 was included in the final models. Y6 is the original search window of the first stage and is the reference window. The RMSE values of the optimization runs carried out in different years, together with their comparison with Y6, are given in Table 6.

The validation RMSE distributions differ significantly across windows according to the Kruskal–Wallis test: H = 76.73; *p* = 8.6 × 10^−16^; and n = 150. Post hoc Mann–Whitney U tests show that each of the Y2–Y5 windows differs significantly from Y6 after Holm correction (Holm-corrected *p*-values are all below 1.3 × 10^−10^). The median search RMSEs of the other windows are between 30.7% and 44.8% higher than the Y6 reference.

In contrast, the deviation between the RMSE values from the final training on the full data is much smaller. The final RMSEs of the Y2–Y5 windows are distributed in the range of 0.08252–0.08550 and deviate from the Y6 reference (0.07993) by at most 7.0%. The inter-window variance observed in the hyperparameter search stage is largely absorbed after retraining on the full data. The practical implication is that a single-year exploration strategy is operationally defensible.

The consistency of best configurations across windows is summarized in Table 7. Among the categorical hyperparameters, the mother wavelet was db4 in 60% of the windows, the fill mode was symmetric in 60%, and the batch size was 32 in 80% of the windows. Among the continuous hyperparameters, the decomposition level shows the lowest coefficient of variation (8.3%). On the other hand, the number of hidden units (CV 47.9%), the number of layers (CV 64.8%) and the dropout rate (CV 57.9%) exhibit low inter-window consistency. This indicates that the BiLSTM architectural hyperparameter space contains multiple regions of comparable quality; in other words, markedly different architectures can achieve similar validation performance.

To provide a genuinely out-of-sample assessment, we additionally performed a rolling-origin experiment for the recommended GWO configuration. This contrasts with the multi-window robustness analysis above, in which each configuration is retrained on the full record. At each origin the model was trained only on past years (with the per-window configuration selected without seeing the test year), the following year was used for early stopping, and a later, completely unseen year was used only for testing, with causal decomposition throughout. For the origin trained on years 1–2, validated on year 3 and tested on the unseen year 4, the reconstructed forecast attains RMSE = 1.007 ± 0.006 m/s over ten training seeds (R^2^ = 0.38), against a persistence baseline of 0.648 m/s on the same test year, corresponding to a skill score of −0.55. Consistent with Section 4.5, the architecture does not exceed naive persistence in a strict out-of-sample setting at the one-hour horizon; the tight seed spread (±0.006 m/s) confirms that this outcome is governed by generalization rather than by training-level randomness.

### 4.4. Component Ablation Studies

In the fourth stage, two ablation scenarios were run on the GWO configuration that won the first stage. These are the “nodwt” runs, in which the decomposition layer is disabled and the raw wind speed signal is fed directly to the BiLSTM, and “nobidir” studies, in which one-way LSTM is used instead of BiLSTM. Both scenarios were run with the seed set of the first stage, which allowed the ablation differences to be characterized with paired Wilcoxon tests. The results are summarized in Table 8.

These results reveal a clear asymmetry between the contributions of the architectural components. When the DWT decomposition layer was disabled, all 30 paired runs produced a higher RMSE than the baseline (Cliff δ = 1.00), and the median validation RMSE increased by 50.8%. The effect size for the decomposition layer is very large (Cohen’s d = 13.09), with *p* = 1.51 × 10^−11^. These results show that the DWT decomposition layer is an essential component of the proposed architecture.

No statistically significant performance decrease was observed in the bidirectional-free scenario. When one-way LSTM was used instead of BiLSTM, the median difference was only −1.1%. The paired Wilcoxon test gave a completely insignificant result with *p* = 0.674. The small values of Cohen’s d = −0.25 and Cliff δ = −0.07 reinforce this insignificance. This result shows that over the one-hour prediction horizon (H = 1) the past sequential context is much more dominant than the future context; thus, bidirectional processing provides a negligible performance contribution at marginal computational cost.

The Friedman test applied for the triple ablation comparison confirms a significant overall difference: χ^2^ = 45.07; df = 2; and *p* = 1.64 × 10^−10^. The average rankings are full = 1.53, nobidir = 1.47 and nodwt = 3.00, confirming that only the removal of DWT produced a significant difference in rank and that the full and nobidir scenarios were statistically indistinguishable.

**Figure 6:**
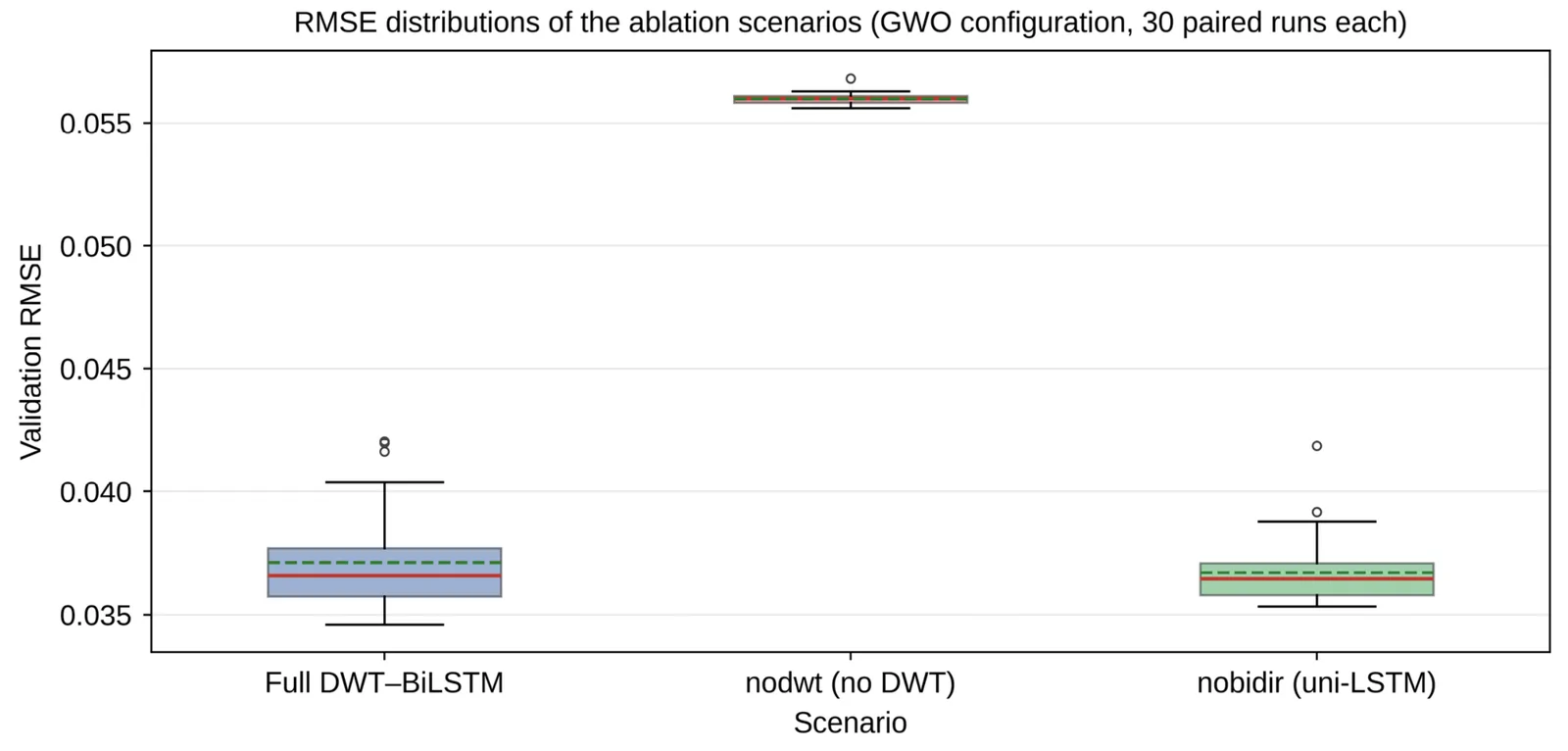
RMSE distributions for HP search validation obtained from 30 independent runs for the baseline (full DWT–BiLSTM), nodwt (DWT layer disabled), and nobidir (unidirectional LSTM instead of BiLSTM) scenarios. In each box, the red solid line is the median, the green dashed line is the mean, the box spans the interquartile range (IQR), the whiskers extend to 1.5× IQR, and open circles are outliers.

Figure 6 visually compares the distributions of 30 runs in ablation studies. Because each scenario was run using equal seed set with Stage 1, the results can be interpreted as paired. While the nodwt scenario exhibits a significant and statistically significant upward shift relative to the baseline (median +50.8%; paired Wilcoxon $p<{1.51\times 10}^{-11}$; Cohen d=13.09), the nobidir scenario is practically indistinguishable from the baseline (p=0.674). The line within the box represents the median, the bottom and top edges of the box represent the first and third quartiles, the “whiskers” represent the 1.5× IQR limit of the distribution, and the circles represent outliers.

A further ablation examined how strongly the results depend on the choice of mother wavelet. Holding the winning BiLSTM configuration, the decomposition level and the fill mode fixed, the wavelet was swept over all twelve candidate families (Daubechies db4–db8, Symlet sym5–sym8, and Coiflet coif3–coif5), with each evaluated under the causal decomposition. Across the twelve families the normalized validation RMSE spanned only 6.0% (from 0.0779 for sym7 to 0.0825 for db7) and the reconstructed one-step RMSE only about 3% (1.011–1.040 m/s); no wavelet allowed the model to surpass the naive persistence baseline of 0.651 m/s. Forecasting performance is thus largely insensitive to the specific wavelet family, indicating that the contribution of the front-end stems from the decomposition itself rather than from a finely tuned basis.

### 4.5. Skill over Naive Persistence

To assess predictive value beyond serial correlation, the GWO-optimized DWT–BiLSTM was compared against a 1-step naive persistence forecast (Section 3.8) on the chronological 20% test partition. Two comparison spaces must be distinguished. In the normalized sub-band space in which the search objective (Equation 1) is defined, persistence yields a mean reference RMSE of 0.1418 ± 0.0505 (eight stations × five sub-bands), against which the GWO model’s component RMSE of 0.0799 corresponds to a nominal skill score of 0.4364. Taken at face value, this suggests the architecture captures structure beyond persistence. However, this normalized, component-averaged quantity is a search surrogate and is not equivalent to the forecasting error of the reconstructed wind speed signal.

We therefore evaluated the reconstructed forecast in physical units. After inverse DWT and inverse scaling, the eight configurations attain a test RMSE of 1.01–1.06 m/s (Table 9), whereas naive persistence attains 0.6510 ± 0.1329 m/s; the reconstructed skill scores are therefore negative (−0.56 to −0.63). In other words, at the one-hour horizon the reconstructed DWT–BiLSTM forecast does not exceed naive persistence. This shortfall is spatially uniform: for the recommended GWO configuration the reconstructed forecast fails to beat the local persistence baseline at every one of the eight stations individually (per-station RMSE 0.86–1.36 m/s versus persistence 0.53–0.88 m/s; skill −0.51 to −0.78), as detailed in Table A2 (Appendix A). A controlled comparison confirms that this is not an artifact of decomposition leakage: repeating the evaluation with a fully causal (train-only) decomposition yields essentially the same result (reconstructed skill −0.59 causal versus −0.54 leaky), consistent with the finding that more than 99.9% of the training-side wavelet coefficients are identical under the two schemes. Reconstructing per-component forecasts of a decimated wavelet transform accumulates error across sub-bands, and one-step persistence is a strong baseline at hourly resolution. We report this transparently: the component-space skill above overstates operational skill, and the value of this study lies in the rigorous, leakage-controlled algorithm benchmark and the evaluation methodology rather than in out-of-sample accuracy at h = 1.

## 5. Discussion

One of the most instructive findings of the first stage is that GA remained third in average performance despite the lowest validation RMSE achieved in a single run (0.0340). In contrast, GWO leads on the mean and standard deviation measures, even though its best single run (0.0346) is not the lowest. This result reflects the stochastic nature of metaheuristic optimization, in which a single run may owe its outcome to the algorithm’s lucky starting point. An unbiased comparison should therefore be based on statistics from repeated runs. The choice of n = 30 runs in the present study represents a defensible lower bound in terms of both classical statistical adequacy and the power of the Friedman and Wilcoxon tests.

The convergence and decision-space-diversity traces (Figure 3 and Figure 4) jointly offer a mechanistic interpretation consistent with GWO’s empirical advantage. GWO’s α–β–δ leader–follower update structure, governed by a linearly decreasing exploration coefficient, is associated with a collapse of the population onto the global best within roughly thirty iterations, after which exploitation continues to refine hyperparameters within the discovered basin. PSO retains a small residual diversity (decay ratio 0.24) because of its inertial momentum, leading to slightly slower convergence but a comparable final loss. ABC’s scout phase, in contrast, periodically reseeds individuals at search-space boundaries every cycle, preventing complete decision-space convergence (decay ratio > 1.0) and explaining why it consistently ranks fourth despite an identical evaluation budget. HO’s bee-cycle interleaving with bounded memory keeps decision-space diversity above 0.19 throughout the run, precluding fine-grained refinement and pushing it to the bottom of the ranking. These observations confirm that, on this nine-dimensional hybrid-architecture problem, fast exploitation rather than persistent exploration is the dominant determinant of solution quality. These diagnostics are correlational rather than causal: they characterize the search dynamics that accompany the observed ranking on this specific problem and are not intended to establish a general mechanistic law.

This study serves as a benchmark by applying the newly proposed FNO [21], Raindrop [22], and TOA [23] methods to wind speed prediction for the first time. These algorithms exhibited average performance that lagged behind the classical metaheuristics. However, their final training RMSEs were close to those of the classical algorithms. This pattern is a direct reflection of the “No Free Lunch” theorem [47]. A general-purpose advantage over a specific problem structure cannot be claimed. The unique mechanisms of these algorithms (FNO’s “further-is-better” reference points, Raindrop’s evaporation–convection dynamics, and TOA’s Tuckman group development stages), despite their performance on synthetic test functions, have failed to compete with the mechanisms of classical algorithms which have been adapted for decades of widespread application in the complex categorical–continuous search space. This limitation may change as the research community develops problem-specific initialization strategies and tunes control parameters for such problems.

The wall-time analysis in Table 4 sharpens the practical recommendation, since GWO dominates on this benchmark. It attains the lowest mean RMSE and simultaneously the second-fastest wall-time (cost ratio 1.09× of the fastest algorithm, FNO). There is therefore no accuracy–cost trade-off to negotiate. ABC, in contrast, consumes 3.67× the wall-time of FNO while delivering uncompetitive accuracy, making it a strict non-recommendation for this problem class regardless of the otherwise low run-to-run dispersion. The new algorithms (FNO, Raindrop, TOA) cluster in an intermediate region. Their wall-time is favorable, but their final RMSE plateau is higher than that of GWO. This outcome suggests that recency alone does not translate into a problem-specific advantage on this nine-dimensional hybrid-architecture search.

Framed against the taxonomic contrast introduced in Section 3.5, the results provide a direct answer to whether biological metaphor confers a search advantage on this problem: it does not, at least not by itself. The three best-ranked algorithms on the repeated-run benchmark are all biologically or evolutionarily inspired (GWO, PSO, GA), yet the biologically inspired Hippopotamus Optimizer ranks last, below the deliberately metaphor-free FNO. In the final full-length training runs the ordering shifts again, with the physically inspired Raindrop Optimizer and the socially inspired Tuckman Optimization Algorithm attaining the lowest RMSE values, narrowly ahead of the classical bio-inspired methods. Taken together, membership in the biological or evolutionary family neither guarantees nor precludes competitive performance here; what separates the algorithms is the fit between their exploration–exploitation balance and the fast-exploitation regime that this nine-dimensional hybrid-architecture landscape rewards, together with the accumulated maturity of control parameters refined over decades of use. For a field in which new animal- and nature-themed metaheuristics continue to proliferate, this benchmark is a reminder that the source of a metaphor is far less informative than its underlying search mechanics and its degree of problem-specific adaptation.

The findings of the second stage indicate that the answer to RQ2 is statistically robust. That joint optimization prevailed in 100% of the 90 equally seeded comparisons across all three algorithms (*p* < 10^−9^, Cliff’s δ = 1.00) is strong evidence that the interaction between decomposition-parameter selection and architecture selection is not negligible in the end-to-end system. We frame this as evidence rather than proof, because the sequential baseline selects decomposition parameters by Shannon entropy minimization rather than by the downstream validation loss (see the Limitations Section). Entropy-based decomposition selection is itself a standard, widely used sequential practice [48], so the baseline is representative of common workflows; because the two schemes optimize different objectives for the decomposition step, however, the comparison does not isolate the effect of joint optimization from that of the selection criterion, and the joint–sequential result is therefore treated as suggestive rather than definitive. A budget-matched bilevel comparison, in which both the decomposition parameters and the BiLSTM architecture are searched against the same downstream validation loss under an equal computational budget, is a natural way to settle this question definitively and is left for future work.

Our study observes that the DWT configuration chosen by Shannon entropy in the sequential configuration (sym6/level = 3/zero) is systematically different from the configurations mostly chosen by the three algorithms in the joint configuration (db4/level = 4/symmetric). This illustrates the difference in purpose between the mathematical compactness of the decomposition metric and the maximization of downstream model performance. A self-defined compact decomposition may not be the most efficient method for learning BiLSTM.

A notable observation from the multi-window robustness analysis is that the inter-window variance of 30–45% in the hyperparameter search RMSEs drops to at most 7% in the final training stage. This pattern concretely quantifies the literature’s doubts [3] that the hyperparameter search metric may differ from the operational distribution metric. The finding demonstrates that the study’s one-year search design is operationally defensible and suggests that the marginal benefits offered by more expensive multi-year search strategies should be weighed against their high computational costs. As each window contributes a single retrained value, this across-window comparison is reported descriptively rather than as a formal significance test; the statistically grounded, seed-replicated out-of-sample assessment is provided by the rolling-origin evaluation of Section 4.3.

Conceptually, this finding parallels the robustness discussions under “distribution shift” in the numerical optimization literature. The flatness of the effective basin in the hyperparameter space makes it possible to smooth out the distributed model performance even if different local minima are reached from different search trajectories. As shown in Table 6 in Section 4.3, while the continuous BiLSTM hyperparameters (hidden, layers, dropout) exhibit a high inter-window coefficient of variation, their final RMSEs cluster within an acceptable range. This result indicates that having strict control over hyperparameter configuration is not critical for average distribution quality. Instead, the adequacy of the data coverage plays a decisive role.

In the interest of scientific transparency, it is noted that different seed values were used for the third stage compared to the first stage. While the windows of the second stage (Y2–Y5) used deterministic seeds (42001–42030), the first stage (Y6) used a set of random seeds. Due to this difference in seeds, the Mann–Whitney U test for independent samples was used instead of a paired *t*-test in the cross-window comparison. However, it is assessed that the sample size of each window, with n = 30 conditions, is sufficient to smooth out the variance at the seed level.

The fourth stage clearly and asymmetrically highlights the contribution of architectural components. Disabling the DWT decomposition layer significantly increased the RMSE in all 30 paired runs (median +50.8%; *p* = 1.51 × 10^−11^; Cohen’s d = 13.09). This represents an enormous effect size in a level rarely encountered in experimental studies according to Cohen’s classification. This finding demonstrates that the DWT decomposition layer is not merely an auxiliary but an essential architectural component, reinforcing the finding by Barjasteh et al. [5] of a 16–26% improvement.

The marginal contribution of bidirectional processing, however, requires a more nuanced discussion. The fact that replacing the BiLSTM with a unidirectional LSTM resulted in a difference of only −1.1% in the median RMSE, and that the paired Wilcoxon test yielded a *p*-value of 0.674, indicates that the bidirectional architecture provides a marginal contribution at a one-hour forecast horizon (H = 1). This finding is important from a practical standpoint. The GWO baseline already uses a compact BiLSTM with a configuration of layers = 1 and hidden = 85. Replacing this architecture with a unidirectional LSTM reduces the number of model parameters by approximately half and offers a design option that significantly lowers computational costs in edge device or IoT deployment contexts.

Taken together, the multi-stage protocol supports the methodological premise of this work: hybrid decomposition–deep-learning systems benefit from treating front-end signal-processing parameters as searchable by bio-inspired metaheuristics rather than fixing them by expert convention, and the joint search is statistically superior to the sequential alternative. We emphasize, however, that this superiority is established in the search and validation space; as shown in Section 4.5, it does not translate into skill over naive persistence once forecasts are reconstructed to physical units at the one-hour horizon. The contribution is therefore methodological. It provides a rigorous, leakage-controlled benchmark and an honest evaluation protocol rather than a claim of state-of-the-art forecasting accuracy.

A broader lesson concerns evaluation practice in the decomposition–forecasting literature. Two design choices are common in this literature: decomposing the full series before the train/test split and reporting errors in the normalized or per-component space. Each can make a method appear to beat persistence when it does not. In our data the first (leakage) proved numerically negligible (more than 99.9% of training coefficients identical to a causal decomposition), but the second was decisive: a component-space skill of +0.44 coexists with a reconstructed m/s skill of −0.55. We therefore recommend that decomposition-based forecasting studies report reconstructed, physical-unit errors against a persistence baseline on an untouched test set, and we adopt this standard here.

This study makes the following contributions to address three gaps identified in the literature. (i) It is the first study to present a systematic comparison of eight algorithms for hyperparameter optimization in the field of wind speed prediction. Previous comprehensive comparisons have been limited to three [11] or four [12] algorithms. (ii) Joint and sequential optimization approaches are compared for the first time using controlled paired experiments on the same problem. (iii) The FNO, Raindrop, and TOA methods are reported for the first time in wind speed prediction and establish a benchmark for future studies.

This study should be reported along with a number of limitations that could affect its validity.

- *Limitation in terms of data coverage*: The study is based on data obtained from eight meteorological stations located in a single geographic region (Nevşehir, Central Anatolia). A multi-region extrapolation study to confirm that the GWO configuration will also perform well under different climate regimes is left for future research.
- *Forecast horizon constraint*: All experiments were conducted with a forecast horizon of H = 1 (one hour ahead). It is assumed that the finding regarding the marginality of bidirectional processing might yield different results at longer forecast horizons (H = 6, 12, 24). This is a topic beyond the scope of this study.
- *Seed protocol restrictions*: Deterministic seeds (42001–42030) were used for the multi-window analysis windows (Y2–Y5), and random seeds were used for Stage 1 (Y6). For this reason, the independent-sample Mann–Whitney U test was applied instead of the paired Wilcoxon test in the statistical tests in Section 4.3.
- *The single-channel nature of the sequential optimization criterion*: In the sequential framework, DWT parameter selection was performed solely using the Shannon entropy minimization criterion. Alternative criteria (e.g., minimum description length, cross-validation-based criteria) may select different decomposition configurations and affect the combined–sequential comparison in different ways. The current results are valid specifically in the context of Shannon entropy.
- *Horizon and reconstruction limitation*: All forecasts are one-step (H = 1) and, when reconstructed to physical units, do not exceed a naive persistence baseline (Section 4.5). This reflects both the strength of persistence at hourly resolution and the error accumulation inherent in reconstructing per-component forecasts of a decimated wavelet transform; longer horizons and shift-invariant (undecimated) transforms are expected to be more favorable and are left for future work.
- *Consideration of the multi-station joint model in isolation*: The current model uses a single global BiLSTM to model eight stations. Station-specific adaptations using federated learning or transfer learning approaches could improve performance. However, this is beyond the scope of this study.

The practical recommendations for operational use presented in this study can be summarized as follows: Using GWO as the hyperparameter search algorithm in DWT–BiLSTM-based wind speed forecasting systems is the recommended starting point. From a data strategy perspective, the combination of single-year searching and full-scale retraining provides both computational savings and operational robustness, and this approach represents a preferable balance since the marginal gain of multi-year searching is offset by the high computational cost. In terms of architectural decisions, while the DWT layer is indispensable, using a unidirectional LSTM instead of BiLSTM reduces the number of model parameters by approximately half while causing a statistically negligible loss in performance, a feature that makes it attractive for IoT and edge device applications. Finally, from an optimization paradigm perspective, the combined architecture is statistically significantly superior to sequential architecture. Therefore, it is recommended that decomposition and model parameters be addressed within the same search space in new hybrid model designs.

## 6. Conclusions

This study systematically compared eight metaheuristic algorithms for joint hyperparameter optimization of a DWT–BiLSTM wind speed forecasting architecture across a unified nine-dimensional search space covering decomposition, architecture, and training parameters. Experiments were conducted over 51,144 h of wind speed observations per station from eight stations in Nevşehir, Türkiye (409,152 total samples), with 30 independent runs per algorithm under a fixed computational budget.

Four main conclusions emerge. First, Grey Wolf Optimizer achieved the best overall performance (mean Friedman rank 2.27; χ^2^ = 49.76, *p* < 10^−8^), followed by PSO and GA. The mean rank metric proved more discriminative than single-run minimum RMSE, highlighting the importance of distribution-based assessment for stochastic optimizers. Second, joint optimization yielded a median validation RMSE approximately 39% lower than the sequential baseline across all three tested algorithms, with Cliff’s δ = 1.00 and *p* < 10^−9^ in each case, confirming that DWT and BiLSTM parameters are strongly interdependent and must be explored simultaneously. Third, the three newly proposed algorithms (TOA, Raindrop, FNO) did not outperform their classical counterparts, a result consistent with the No Free Lunch theorem. Fourth, multi-window robustness analysis on GWO across five annual windows demonstrated that hyperparameter search outcomes varied significantly across windows (Kruskal–Wallis $p<10^{-15}$), yet final retrained model RMSEs converged to within 7% of the reference window, confirming the robustness of the proposed framework. The algorithm ranking reported here is specific to the DWT-BiLSTM architecture and the nine-dimensional joint search it induces and should be read as architecture-specific rather than universal; whether the same ordering holds for other forecasting backbones, such as Transformer-, temporal-convolutional- (TCN) and GRU-based models, is left for future work.

Component ablation further revealed that DWT preprocessing is a crucial element. The experiments showed that removing it increased the median RMSE by 50.8% (Cohen’s *d* = 13.09, *p* = 1.51 × 10^−11^), whereas replacing BiLSTM with a unidirectional LSTM produced no statistically significant change (*p* = 0.674). Building on these results, we identify four concrete directions for future work: (i) evaluating a broader pool of optimizers, including large-scale and hybrid or memetic metaheuristics, beyond the eight compared here; (ii) validating the framework on broader, multi-region benchmark datasets spanning different climate regimes; (iii) extending the evaluation to multi-step forecast horizons and to a shift-invariant (undecimated) wavelet transform; and (iv) assessing real-world operational deployment, including edge and IoT execution and integration with federated- and transfer-learning schemes for station-specific adaptation.

Finally, an honest evaluation caveat frames these results: while the algorithm comparison and the component ablation are robust, evaluating the reconstructed forecast in physical units shows that the optimized architecture does not surpass naive persistence at the one-hour horizon (Section 4.5). We therefore present this work as a rigorous, leakage-controlled benchmark of bio-inspired metaheuristics and an honest evaluation methodology. Extending the architecture to longer forecast horizons and to a shift-invariant (undecimated) wavelet transform is left for future work. These are settings in which decomposition-based forecasting is more likely to add value over persistence.

## Figures and Tables

**Table 1:** Comparison of representative studies in terms of optimization scope.

Study	Area	Decomposition	Model	Algorithm Number	Search Scope	Optimization	Statistical Test
Sen et al. [11]	Weather (temperature)	No decomposition	ANN/LSTM/GRU	3	Model HP (architecture)	Only model	—
El-kenawy et al. [13]	Wind speed	No decomposition	MLP + KNR + LSTM (ensemble)	6	Feature selection + ensemble weights	Only model	ANOVA, Wilcoxon
Alharbi et al. [12]	HVAC energy	No decomposition	BiLSTM	10	~5–6D (units, layers, LR, batch, dropout, epochs)	Only model	—
Hanifi et al. [3]	Wind power	No decomposition	CNN/LSTM	3	~5–6D (architecture + training)	Only model	—
Amirteimoury et al. [4]	Wind speed	DWT	BiLSTM	1	1 D (MI threshold)	Only decomposition	—
Li et al. [14]	Wind speed	VMD	BiLSTM-A	2	Decomp (GA) + model HP (IDBO)	Sequential	—
Zhang et al. [9]	Wind speed	VMD	TCN + Transformer	1	2 D (decomposition)	Only decomposition	DM test
Gu et al. [25]	Wind power	SVMD	TCN + BiGRU	4	~5 D (model)	Only model	—
This study	Wind speed	DWT	BiLSTM	8	9 D (decomposition + model + training)	Joint 9D	Friedman, Wilcoxon, Holm, effect size

**Table 2:** Nine dimensions of the joint search space.

No	Parameters	Range/Options	Type	Scale	Layer
1	Mother wavelet	db4–db8, sym5–sym8, coif3–coif5 (12 options)	Categoric	—	Decomposition
2	Decomposition level (*L*)	3–7	Integer	Linear	Decomposition
3	Fill mode	Symmetric, zero, periodization	Categoric	—	Decomposition
4	Look-back (*w*)	24–96 Hours	Integer	Linear	Data representation
5	Number of hidden units	32–256	Integer	Linear	Model
6	Number of layers	1–4	Integer	Linear	Model
7	Dropout rate	0.0–0.5	Continuous	Linear	Model
8	Learning rate	10^−4^–10^−2^	Continuous	Logarithmic	Training
9	Batch size	32, 64, 128	Categoric	—	Training

**Table 3:** DWT–BiLSTM combined optimization performance of eight metaheuristic algorithms (30 independent runs/algorithm).

Rank	Algorithm	Min	Average	Median	Std.	CV (%)	Mean Rank	Final RMSE
1	**GWO**	0.03458	**0.03712**	**0.03656**	**0.00199**	5.37	**2.27**	0.07993
2	PSO	0.03461	0.03824	0.03734	0.00319	8.35	2.97	0.07966
3	GA	**0.03401**	0.03916	0.03766	0.00343	8.76	4.00	0.08153
4	ABC	0.03737	0.03990	0.03973	0.00176	4.40	4.37	0.07896
5	TOA	0.03662	0.04006	0.03935	0.00221	5.51	4.60	0.07822
6	Raindrop	0.03705	0.04110	0.04136	0.00201	4.89	5.23	0.07819
7	FNO	0.03657	0.04212	0.04294	0.00220	5.23	6.27	0.07933
8	HO	0.03635	0.04224	0.04282	0.00238	5.64	6.30	0.07899

Bold indicates the best value in each column.

**Table 4:** Wall-time cost per metaheuristic algorithm (30 runs each, 40 individuals × 50 iterations = 2000 surrogate evaluations per run). Cost ratio is relative to the fastest algorithm (FNO).

Rank	Algorithm	Mean Total (h)	SD (h)	Mean per Iter (s)	Cost Ratio
1	FNO	19.7	5.9	1422	1.00×
2	GWO	21.6	15.5	1553	1.09×
3	GA	23.5	16.0	1694	1.19×
4	PSO	24.0	18.8	1728	1.22×
5	Raindrop	24.3	3.6	1747	1.23×
6	HO	25.9	3.2	1864	1.31×
7	TOA	27.5	2.8	1983	1.40×
8	ABC	72.5	6.2	5221	3.67×

**Table 5:** Paired run comparison of joint and sequential optimization (3 algorithms x 30 paired runs).

Algorithm	Joint Median	Sequential Median	Joint Avg.	Sequential Avg.	Wilcoxon Signed-Rank Test *p*	Cliff Delta
GWO	0.03656	0.06088	0.03712	0.06133	<10^−9^	1.00
PSO	0.03734	0.06125	0.03824	0.06227	<10^−9^	1.00
GA	0.03766	0.06106	0.03916	0.06157	<10^−9^	1.00

**Table 6:** Window-based hyperparameter search distribution (30 runs) and final training RMSE for GWO.

Window	Search Median	Search Avg.	Search Std.	Final RMSE	Δ vs. Y6 (Search)	Δ vs. Y6 (Final)
Y2	0.05016	0.05014	0.00397	0.08252	+37.2%	+3.2%
Y3	0.04929	0.04843	0.00461	0.08493	+34.8%	+6.3%
Y4	0.05292	0.05164	0.00495	0.08550	+44.8%	+7.0%
Y5	0.04780	0.04918	0.00510	0.08437	+30.7%	+5.6%
Y6	**0.03656**	**0.03712**	**0.00199**	**0.07993**	**reference**	**reference**

Bold indicates the best value in each column.

**Table 7:** Hyperparameter consistency across window conditions.

Hyperparameter	Type	Modal Value/Average	Modal Frequency/CV (%)	Comment
Mother wavelet	Categoric	db4	60% (3/5 window)	Consistent
Fill mode	Categoric	Symmetric	60% (3/5 window)	Consistent
Batch size	Categoric	32	80% (4/5 window)	Very consistent
Decomposition level (*L*)	Integer	4.80	8.3% (CV)	Very consistent
Look-back (*w*)	Integer	61.4 h	24.1% (CV)	Moderately consistency
Learning rate (*lr*)	Continuous	7.4 × 10^−3^	25.0% (CV)	Moderately consistency
Number of hidden units	Integer	126.8	47.9% (CV)	Low consistency
Dropout rate	Continuous	0.244	57.9% (CV)	Low consistency
Number of layers	Integer	1.80	64.8% (CV)	Low consistency

**Table 8:** Comparison of ablation scenarios with GWO baseline (30 paired runs). * < 0.05 threshold; statistically significant.

Scenario	Median RMSE	Δ vs. Baseline	Wilcoxon *p*	Cohen d	Cliff δ
Baseline (DWT + BiLSTM)	0.03656	reference	—	—	—
nodwt	0.05599	+50.8%	1.51 × 10^−11^	13.09 (major impact)	1.00 (major impact)
nobidir	0.03644	−1.1%	0.674	−0.25 (small impact)	−0.07 (small impact)

**Table 9:** Reconstructed one-step (h = 1) forecast performance in physical units for the eight best configurations under the causal evaluation, aggregated over the eight stations, with the persistence-relative skill score (persistence RMSE = 0.651 m/s). All skill scores are negative.

Algorithm (Wavelet)	RMSE (m/s)	MAE (m/s)	R^2^	nRMSE	MAPE (%)	Skill
FNO (sym6)	1.015	0.724	0.44	0.128	41.3	−0.56
ABC (sym6)	1.016	0.727	0.44	0.128	42.0	−0.56
TOA (coif4)	1.024	0.724	0.43	0.129	41.4	−0.57
Raindrop (db5)	1.028	0.734	0.43	0.130	42.2	−0.58
PSO (sym6)	1.035	0.742	0.42	0.131	43.4	−0.59
GWO (db4)	1.038	0.735	0.41	0.131	42.0	−0.60
HO (db5)	1.040	0.741	0.41	0.131	43.2	−0.60
GA (coif4)	1.059	0.754	0.39	0.134	43.3	−0.63

## Data Availability

The analysis code, configuration files, per-iteration trial-history databases, and aggregated result files that support the findings of this study are openly available on GitHub (https://github.com/emrebendes/wind-dwt-bilstm-metaheuristic, accessed on 4 August 2026) and archived on Zenodo (https://zenodo.org/records/21384094). The raw hourly wind speed series are subject to the data-sharing terms of the Turkish State Meteorological Service (MGM) and are not redistributed; they may be requested from MGM at https://mgm.gov.tr, (accessed on 4 August 2026).

## Appendix A

Table A1 reports the control-parameter settings of the eight optimizers. Each value is the default recommended in the algorithm’s original publication; no algorithm-specific tuning was performed, and all methods shared the same search budget (population 40, 50 iterations, 2000 objective evaluations) over the same nine-dimensional unit search space.

**Table A1:** Control-parameter settings of the eight compared optimizers.

Algorithm	Control Parameters (Original-Paper Defaults)
ABC	abandonment limit = 5; equal employed and onlooker bee populations
GA	crossover rate = 0.8; mutation rate = 0.1; tournament size = 3; elitism count = 2; SBX distribution index = 20; polynomial-mutation index = 20
PSO	inertia weight w decreased linearly 0.9 to 0.4; cognitive coefficient c1 = 2.0; social coefficient c2 = 2.0; velocity clamp = 0.2
GWO	encircling coefficient a decreased linearly 2.0 to 0; A = 2a × r1−a; C = 2 × r2
HO	phase population ratios 0.50/0.30/0.20 (river-and-pond/defensive/evasion)
FNO	exploration ratio = 0.5; dynamic-focus shrink factor = 0.7; step size in [0.1, 0.6]
Raindrop	splash radius = 0.15; evaporation threshold = 0.7 (fitness percentile); evaporation rate = 0.1
TOA	sub-team sizes {3, 4, 5}; coordination-index scale = 0.2; logistic growth k_max = 1.0

**Table A2:** Per-station reconstructed one-step (h = 1) forecast metrics in m/s for the recommended configuration (GWO) on the untouched test set, with the per-station persistence baseline and persistence-relative skill. Values are means over three random seeds; per-seed standard deviations are below 0.01 m/s for RMSE. Skill is negative at every station.

Station	RMSE (m/s)	MAE (m/s)	R^2^	nRMSE	Persistence RMSE (m/s)	Skill
S1	0.861	0.630	0.372	0.125	0.525	−0.641
S2	1.313	0.917	0.392	0.154	0.836	−0.570
S3	1.082	0.781	0.365	0.139	0.719	−0.505
S4	0.877	0.613	0.424	0.124	0.558	−0.571
S5	0.952	0.669	0.449	0.121	0.535	−0.779
S6	0.939	0.636	0.463	0.129	0.624	−0.505
S7	0.881	0.645	0.449	0.119	0.535	−0.646
S8	1.355	0.936	0.443	0.134	0.875	−0.548
Mean	1.033	0.716	0.420	0.131	0.651	−0.596
